# PTX3 modulates the immunoflogosis in tumor microenvironment and is a prognostic factor for patients with clear cell renal cell carcinoma

**DOI:** 10.18632/aging.103169

**Published:** 2020-04-28

**Authors:** Giuseppe Stefano Netti, Giuseppe Lucarelli, Federica Spadaccino, Giuseppe Castellano, Margherita Gigante, Chiara Divella, Maria Teresa Rocchetti, Federica Rascio, Vito Mancini, Giovanni Stallone, Giuseppe Carrieri, Loreto Gesualdo, Michele Battaglia, Elena Ranieri

**Affiliations:** 1 Clinical Pathology Unit and Center of Molecular Medicine, Department of Medical and Surgical Sciences, University of Foggia, Viale Luigi Pinto 71122, Foggia, Italy; 2 Urology and Renal Transplantation Unit, Department of Emergency and Organ Transplantation, University of Bari “Aldo Moro”, Bari 70124, Italy; 3 Nephrology Dialysis and Transplantation Unit, Department of Emergency and Organ Transplantation, University of Bari “Aldo Moro”, Bari 70124, Italy; 4 Nephrology Dialysis and Transplantation Unit, Department of Medical and Surgical Sciences, University of Foggia, Viale Luigi Pinto 71122, Foggia, Italy; 5 Urology and Renal Transplantation Unit, Department of Medical and Surgical Sciences, University of Foggia, Viale Luigi Pinto 71122, Foggia, Italy

**Keywords:** renal cell carcinoma, pentraxin 3, complement system, biomarker

## Abstract

Pentraxin-3 (PTX3) belongs to the pentraxine family, innate immune regulators involved in angiogenesis, proliferation and immune escape in cancer. Here, we evaluated PTX3 tissue expression and serum levels as biomarkers of clear cell renal cell carcinoma (ccRCC) and analyzed the possible role of complement system activation on tumor site. A 10-year retrospective cohort study including patients undergoing nephrectomy for ccRCC was also performed. PTX3 expression was elevated in both neoplastic renal cell lines and tissues, while it was absent in both normal renal proximal tubular cells (HK2) and normal renal tissues. Analysis of complement system activation on tumor tissues showed the co-expression of PTX3 with C1q, C3aR, C5R1 and CD59, but not with C5b-9 terminal complex. RCC patients showed higher serum PTX3 levels as compared to non-neoplastic patients (p<0.0001). Higher PTX3 serum levels were observed in patients with higher Fuhrman grade (p<0.01), lymph node (p<0.0001), and visceral metastases (p<0.001). Patients with higher PTX3 levels also showed significantly lower survival rates (p=0.002). Our results suggest that expression of PTX3 can affect the immunoflogosis in the ccRCC microenvironment, by activating the classical pathway of CS (C1q) and releasing pro-angiogenic factors (C3a, C5a). The up-regulation of CD59 also inhibits the complement-mediated cellular lysis.

## INTRODUCTION

Renal cell carcinoma (RCC) is the most common type of renal neoplasia and accounts for about 3% of all adult malignancies in western countries [1]. Recent estimates have calculated that in 2020, in the United States 73,750 new cases will be diagnosed and 14,830 patients will die of RCC [2]. Due to usually asymptomatic clinical course, the diagnosis of most cases of renal cancer is often incidental, following diagnostic tests performed for other clinical conditions, and not rarely shows neoplasms in advanced clinical stage [1]. Furthermore, RCC is a chemo- and radio-resistant neoplasia, therefore the current therapeutic strategies are ultimately based on the surgical approach [3].

RCC encompasses a heterogeneous group of cancers derived from renal tubular epithelial cells [4]. The most common renal cancer type in adults is adenocarcinoma: it accounts almost 90% and in a small percentage (2%) it may also be bilateral. Clear cell renal cell carcinoma (ccRCC) is the most frequently diagnosed subtype and causes the most clinically severe phenotype. In addition, up to 30% of the patients present metastatic disease at diagnosis, and around 20-30% of subjects undergoing surgery will suffer recurrence. In this scenario, early diagnosis is crucial for improving the survival rate of these patients, and the introduction of high-throughput omics technologies has led not only to a detailed molecular characterization of RCC, but also to the identification of novel biomarkers [5–13].

In the last few years, several studies have showed that cigarette smoking, obesity, hypertension, diabetes and End Stage Renal Disease, represent common risk factors for this tumor [14–18]. Moreover, an in-depth understanding of the molecular basis of RCC has led to introduction in clinical practice of novel targeted therapies, including anti-angiogenic agents (sorafenib, sunitinib, pazopanib, axitinib, and bevacizumab), mTOR (temsirolimus and everolimus), and immune checkpoint inhibitors (nivolumab). However, these drugs yield partial responses in a minority of patients, with no evidence of complete responses [3, 19].

Modifications of the tumor microenvironment represent a growing field of investigation in order to highlight potential mechanisms of tumor progression and resistance to targeted therapies [20].

In particular during the oncogenesis, the reactive immunoflogosis seems to play a crucial role to counteract the development of neoplastic cells. On the other hand, its persistence may paradoxically promote the cancer progression, by enriching the tumor microenvironment with pro-inflammatory cytokines and growth factors that can lead to uncontrolled proliferative response [21–23]. In this setting Pentraxin-3 (PTX-3) might play a crucial role. PTX3 is an opsonin belonging to the pentraxin superfamily, which acts as pattern recognition molecule (PRM) of the immune system. This molecule is able to recognize microbial fractions and cellular debris, to promote phagocytosis (opsonization), to activate the complement system and to modulate the inflammation process [24].

The similarity of PTX3 with C-reactive protein (CRP), the most widely used inflammation biomarker, has led to investigate the role of PTX3 in several infectious and inflammatory disorders. PTX3 acts as an acute phase protein and its plasma levels increase rapidly (peak at 6-8 hours) from very low baseline values in healthy subjects, up to higher serum levels in inflammatory conditions. PTX3 can be produced directly within the site of inflammation from both resident and infiltrating cell types and acts with a paracrine effect, unlike other short pentraxins (including CRP), which are produced by the liver and released in the blood [23].

Recent studies have shown how PTX3 can influence the pathogenesis of different cancer types, but plays an ambivalent role, i.e. acting as a tumor suppressor or pro-oncogenetic factor in relation to the neoplastic type and the tumor microenvironment. In some cases PTX3 acts as tumor suppressor, inhibiting the proliferation and angiogenesis of FGF2-mediated tumor cells, as well as the epithelial-to-mesenchymal transition (EMT) and its metastatic potential. In other cancer types the high intratumoral expression of PTX3 is associated with poor prognosis [25, 26].

Moreover, PTX3 shares with the other short classic pentraxins the ability to modulate the complement system, through a direct interaction with the key molecules involved in the activation and/or regulation of the complement system cascade. PTX3 mediates the activation of the classical pathway and lectin pathway because of binding with C1q and MBL, and is able to affect the alternative pathway via CFH binding. The complement system, as part of the innate immune system, enhances the ability of antibodies and phagocytic cells to clear microbes and damaged cells from an organism, promotes inflammation, and attacks the pathogen's cell membrane. In the inflammatory context, complement system releases very strong pro-inflammatory molecules, such as the anaphylatoxins C3a and C5a. However, recent evidence show that complement system may promote cell proliferation and regeneration, thus suggesting a possible relationship between complement system activation and cancer [27].

In the present study, we evaluated PTX3 tissue expression and serum levels as biomarkers of clear cell renal cell carcinoma (ccRCC) and analyzed the possible role of complement system activation on tumor site.

## RESULTS

### Gene set enrichment analysis (GSEA) and microarray analysis

**Figure 1:**
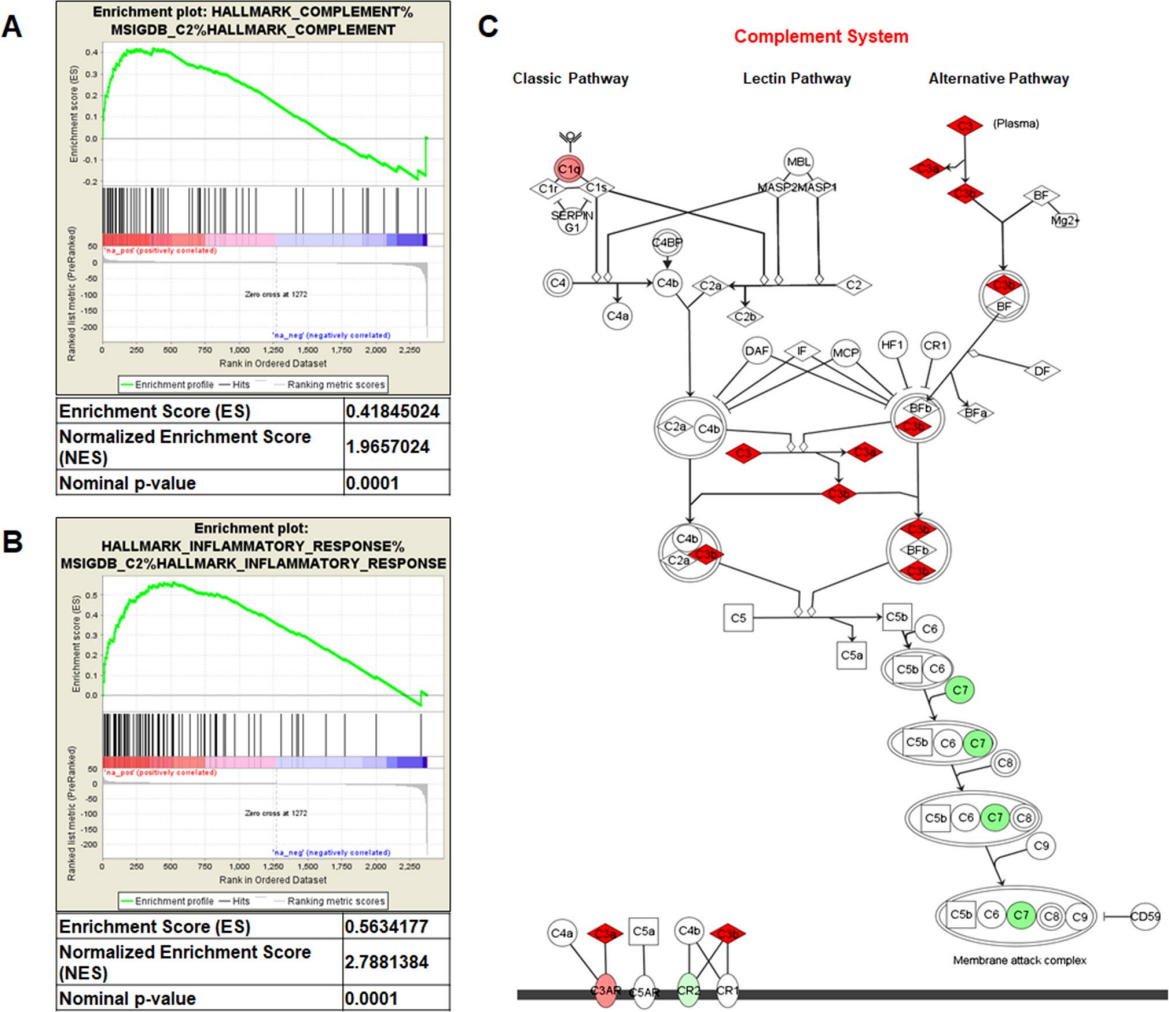
Gene Set Enrichment Analysis (GSEA) of the GSE47032 dataset (**A** and **B**). Complement system pathway from Ingenuity Pathway Analysis (**C**). Genes in green and red are respectively under- and over-expressed in the ccRCC-gene signature.

Gene Set Enrichment Analysis (GSEA) of the GSE47032 dataset showed that ccRCC featured multiple enriched gene sets depicting inflammatory response (NES=2.78; *p*=0.0001; Figure 1A) and complement activation (NES=1.96; *p*=0.0001; Figure 1B). Moreover, microarray analysis using IPA, showed that the canonical molecules associated with complement system were significantly modulated (Figure 1C).

### PTX3 protein expression in RCC cell lines and renal tissues from ccRCC patients

**Figure 2:**
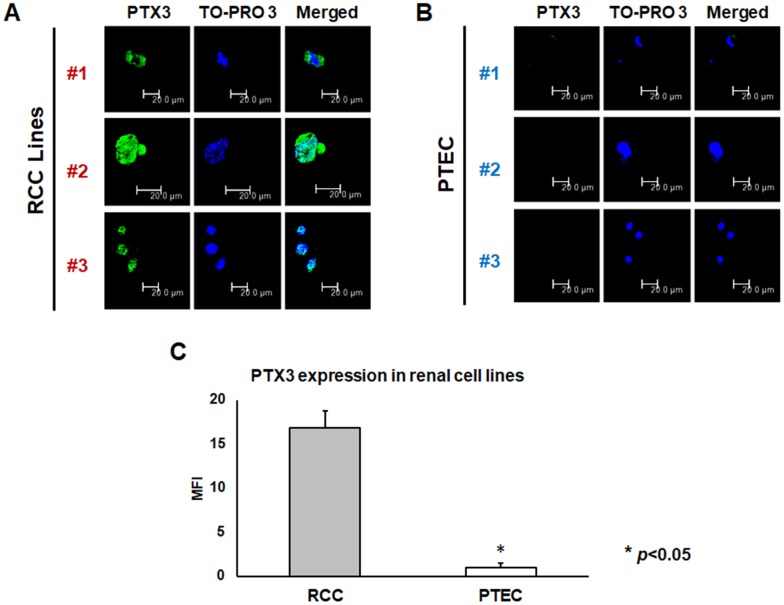
PTX3 expression in neoplastic (**A**) and proximal tubular epithelial cells (PTEC) (**B**) by confocal microscopy and quantification of specific fluorescence (**C**)

PTX3 protein expression was analyzed both in neoplastic and in normal renal proximal tubular epithelial cells (PTEC) by confocal microscopy. Noteworthy, three different renal cancer cell lines showed significantly higher PTX3 expression, as compared by PTEC (Figure 2A, 2B). This observation was strengthened by quantification of specific fluorescence (*p*<0.05; Figure 2C).

Then, we analyzed the tissue expression of PTX3 in tumor tissues of 30 consecutive patients who underwent radical nephrectomy for ccRCC. A control group of 10 subject who underwent renal biopsy in suspicion of chronic nephropathy, but with a normal renal histology, were also analyzed. The main clinical and histologic features of the entire study population of 30 patients with renal clear cell carcinoma subjected to PTX3 tissue expression analyses are summarized in Supplementary Table 1.

**Figure 3:**
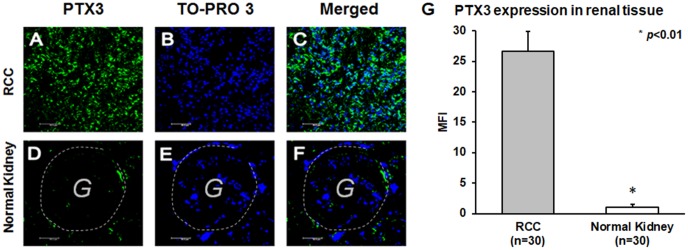
PTX3 expression in neoplastic (**A**-**C**) and normal renal tissues (**D**-**F**) by confocal microscopy and quantification of specific fluorescence (**G**).

PTX3 tissue expression was significantly higher in ccRCC patients (Figure 3A–3C), while in normal kidney it was virtually absent (Figure 3D–3F). This observation was strengthened by quantification of specific fluorescence (*p*<0.01; Figure 3G).

### Complement system activation in renal tissues from ccRCC patients

**Figure 4:**
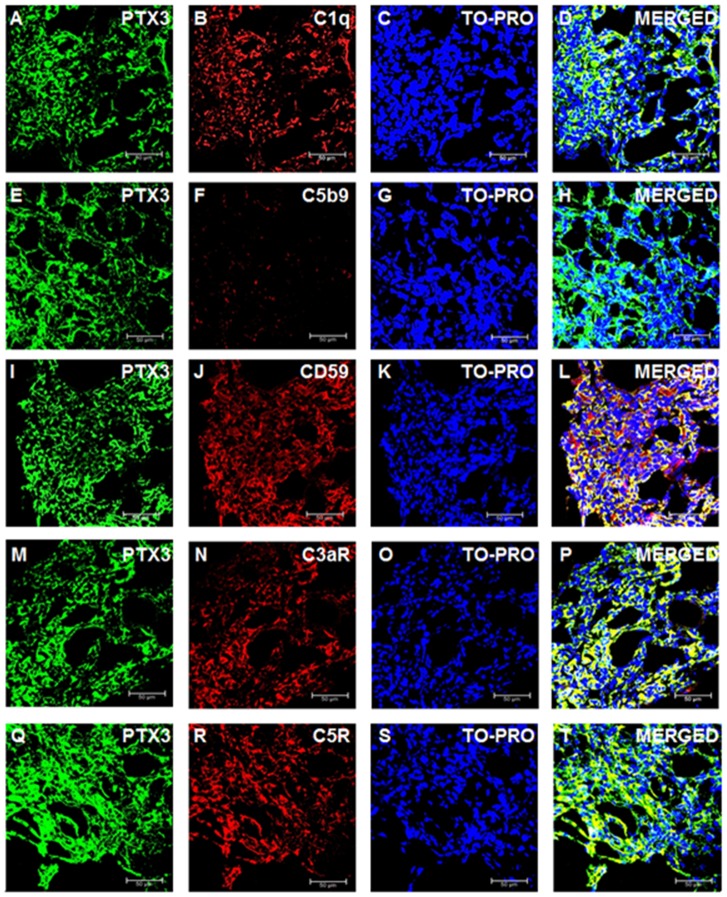
**Complement system factors’ expression and co-localization with PTX3 in renal clear cell carcinoma.** Intra-tumoral expression of PTX3 (green) and co-localization with C1q (**A**-**D**), C5b9 (**E**-**H**), CD59 (**I**-**L**), C3aR (**M**-**P**), C5R (**Q**-**T**).

We then investigated the activation of the complement cascade in renal cancer and in normal renal tissue. Since PTX3 can activate the complement system through the classic pathway, we evaluated the deposition of C1q Interestingly, C1q deposition was extensively present in ccRCC tissue samples and co-localized with PTX3 (Figure 4A–4D). On the other hand, the deposition of MBL, the first protein in the lectin pathway of complement cascade activation, was absent in ccRCC (data not shown).

To validate the complete activation of the complement cascade, we next evaluated the tissue deposition of the terminal complement complex, C5b-9. Surprisingly, the increased activation of the classical pathway of the complement cascade did not correspond to an increased deposition of the terminal complement complex. Indeed, C5b-9 specific immunofluorescence was completely absent in the renal cancer tissue (Figure 4E–4H).

The complement system is characterized by several regulatory proteins that can inhibit the activation of the enzymatic cascade at different levels [26]. CD59 is one of such inhibitors that can prevent C5b-9 assembly [28]. Interestingly, an increased CD59 expression has been reported in several neoplasia [29], although no information is available on the level of CD59 expression within neoplastic renal tissue. Noteworthy, CD59 protein expression was markedly increased in RCC (Figure 4I–4L). Since anaphylotoxins were suggested as possible soluble mediators modulating both cancer cell proliferation and neoplastic angiogenesis [30], we investigated the protein expression of C3a and C5a receptors. The expression of both trans-membrane proteins was markedly increased in ccRCC tissues (Figure 4M–4P and 4Q–4T). Remarkably, the expression of CD59, C3aR and C5aR co-localized with PTX3 expression within the renal cancer tissue samples (Figures 4L, 4P and 4T, respectively).

### PTX3 serum levels as biomarkers of ccRCC

In the attempt to validate the role of PTX3 as a potential biomarker of renal cancer, we retrospectively investigated baseline serum levels of PTX3 in a cohort of 168 consecutive patients undergoing nephrectomy for ccRCC.

The main clinical features of the entire study population of 168 RCC patients, as well as the pathological features of cancers diagnosed are summarized in Table 1.

**Table 1:** Clinical and pathological characteristics of patients who underwent radical or partial nephrectomy for ccRCC.

**Variable**	**n=168**
Age (years)	
median (range)	62 (26-85)
Gender, n (%)	
Male	110 (65.5%)
Female	58 (34.5%)
Dimensions (cm)	
median (range)	5.0 (3 -12)
Pathological stage (TNM/AJCC), n (%)	
pT1	104 (62%)
pT2	24 (14%)
pT3	35 (21%)
pT4	5 (3%)
pN+	34 (20.2%)
cM+	30 (17.8%)
Fuhrman grade, n (%)	
G1-2	101 (60%)
G3-4	67 (40%)

Abbreviations: TNM/AJCC: Tumor size, Lymph Nodes affected, Metastases/American Joint Committee on Cancer.

**Figure 5:**
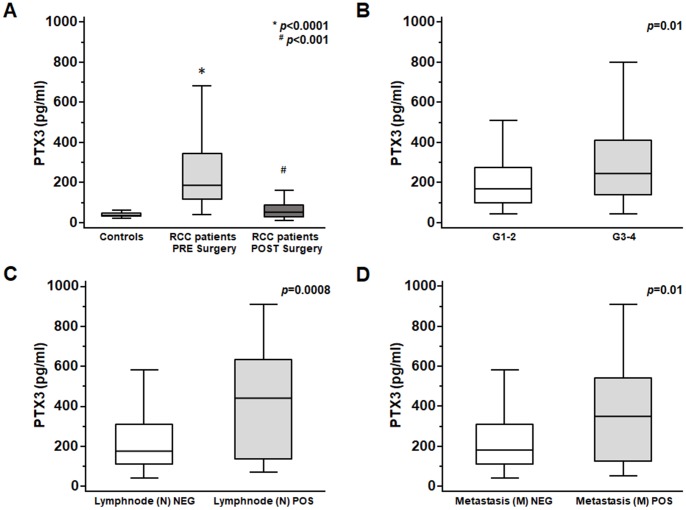
PTX3 serum levels at baseline in patients with renal clear cell carcinoma before and after surgery (**A**) and at different Furhman grading (**B**), lymphnode involvement (**C**) and metastasis staging (**D**).

At time of nephrectomy, PTX3 serum levels were significantly higher in patients with ccRCC as compared with non-neoplastic patients (p<0.0001; Figure 5A).

Moreover, at time of diagnosis significantly higher PTX3 serum levels were observed in patients with higher Fuhrman grade (G3-4 vs G1-2 p<0.01; Figure 5B), with lymph node involvement (N1 vs N0 p<0.0001; Figure 5C), and with visceral metastases (M1 vs M0 p<0.001; Figure 5D).

A ROC curve analysis was carried out to further validate the association of PTX3 serum levels with the cancer-specific survival. The analysis showed that PTX3 serum levels were significantly associated with ccRCC-specific survival (AUC:0.83, p<0.0001) and identified a cutoff value of 165.0 pg/mL with an 86% (95%CI: 73.3-94.2) specificity and a 70.7% (95%CI: 60.7-79.4) sensitivity.

**Figure 6:**
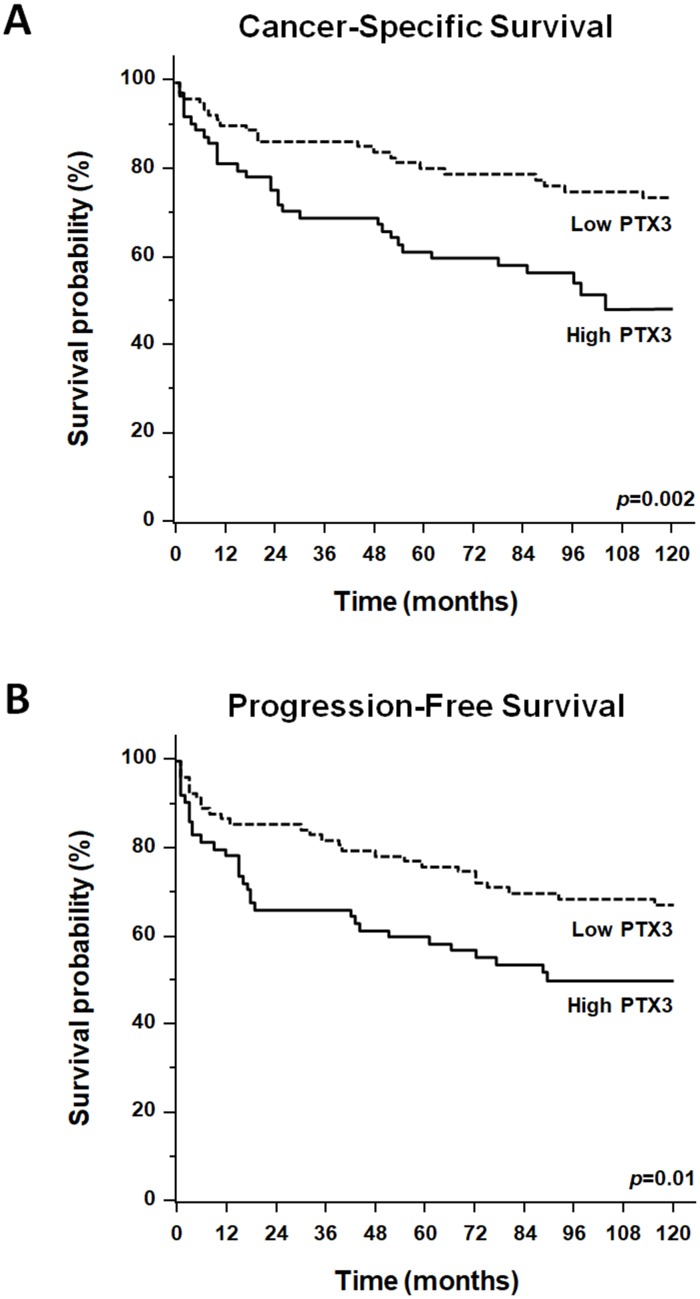
Kaplan-Meier estimate of 12-year cancer-specific survival (CSS: **A**) and progression-free survival (PFS: **B**) of ccRCC patients according to different PTX3 serum levels at baseline.

Survival analysis was performed after the assignment of all patients to two groups according to the operational cut-off of PTX3. ccRCC patients with baseline serum PTX3 levels <165.0 pg/ml showed significantly higher 10-year rate of overall survival, as compared with ccRCC patients with serum PTX3 levels >165.0 pg/ml (73.7% vs. 48.4%, p=0.002; Figure 6).

To estimate the relative risk for cancer-specific survival (CSS) and progression-free survival in ccRCC patients showing serum PTX3 level above or below the operational cut-off, Cox regression analyses were performed using cancer-related death and recurrence as dependent variables, and T stage (T3-4 vs. T1-2), lymph node invasion (N+ vs N0), metastatic disease (M+ vs M0), Furhman grade (G1-2 vs. G3-4), tumor necrosis and size, and PTX3 serum levels as covariates (Table 2).

**Table 2:** Univariate and multivariate analyses for cancer-specific survival.

		**Univariate analysis**					**Multivariate analysis**			
		**CI 95%**					**CI 95%**			
**Variable**	**Category**	**HR**	**Lower**	**Higher**	***p* value**		**HR**	**Lower**	**Higher**	***p* value**
T stage	T3-4 vs T1-2	2.09	1.59	2.75	***0.0001***		1.56	1.14	2.13	***0.004***
N stage	N+ vs N0	3.49	1.84	6.60	***0.001***		1.25	1.01	2.68	***0.02***
M stage	M+ vs M0	6.15	3.44	10.97	***0.0001***		4.77	2.05	9.12	***0.003***
Grade	G3-4 vs G1-2	2.24	1.15	6.86	***0.01***		1.41	1.05	2.08	***0.02***
Necrosis	Yes vs No	2.06	1.18	3.86	***0.01***		-	-	-	***-***
Tumor size	Continuous	1.48	1.12	2.26	***0.01***		-	-	-	***-***
PTX3	>165.0 vs <165.0 pg/ml	2.41	1.21	6.31	***0.001***		1.86	1.05	2.86	***0.01***

CI: confidence interval; HR: hazard ratio.

KaplanMeier survival curves for cancer-specific survival (CSS) and progression-free survival (PFS), stratified by PTX3 serum levels, are shown in Figure 6. Both CSS and PFS were significantly decreased in patients with high levels of PTX3. Univariate analysis for the predefined variables showed that the pathological stage, presence of nodal and visceral metastases, Fuhrman grade, presence of necrosis, tumor size, and high levels of PTX3, were significantly associated with the risk of death (Table 2) and progression (Table 3). At multivariate analysis by Cox regression modeling, the pathological stage, presence of nodal and visceral metastases, Fuhrman grade, and high serum levels of PTX3, were independent adverse prognostic factors for CSS and PFS (Tables 2 and 3).

**Table 3:** Univariate and multivariate analyses for progression-free survival.

		**Univariate analysis**					**Multivariate analysis**			
		**CI 95%**				**CI 95%**			
**Variable**	**Category**	**HR**	**Lower**	**Higher**	***p* value**		**HR**	**Lower**	**Higher**	***p* value**
T stage	T3-4 vs T1-2	2.27	1.76	2.93	***0.0001***		1.60	1.19	2.16	***0.001***
N stage	N+ vs N0	2.38	1.96	7.24	***0.001***		1.22	1.02	2.74	***0.03***
M stage	M+ vs M0	7.19	4.33	12.87	***0.0001***		5.26	2.44	11.42	***0.001***
Grade	G3-4 vs G1-2	2.05	1.54	2.73	***0.01***		1.51	1.09	2.11	***0.01***
Necrosis	Yes vs No	1.95	1.08	3.34	***0.01***		-	-	-	***-***
Tumor size	Continuous	1.34	1.02	2.96	***0.01***		-	-	-	***-***
PTX3	>165.0 vs <165.0 pg/ml	2.01	1.31	7.41	***0.01***		1.56	1.03	2.95	***0.01***

CI: confidence interval; HR: hazard ratio.

## DISCUSSION

The results of the present study suggest that serum concentration of PTX3, a recognized modulator of complement system cascade, might represent a reliable non-invasive biomarker for the diagnosis and prognosis of renal cancer. Moreover, we show that an increased production of PTX3 within the renal tumour may modulate the immunoflogosis in the ccRCC microenvironment, by partially activating the classical pathway of complement system (C1q) and releasing pro-angiogenic factors (C3a, C5a), but inhibiting the complement-mediated cellular lysis due to local up-regulation of CD59.

We showed for the first time an increased expression of PTX3 on both renal cancer cells and ccRCC-derived tissues. Several reports analyzing PTX3 overexpressing cells have suggested that this long pentraxin may exert a pro-tumorigenic role by promoting tumor cell migration and invasion (cervical cancer, head and neck tumors) or proliferation (glioma), epithelial-to-mesenchymal transition (hepatocellular carcinoma) and macrophage chemotaxis [31–34]. PTX3 was also found to be an oncogenic phosphoinositide 3-kinase signaling critical target, involved in promotion of stem cell-like traits in basal-like breast cancers [35].

In previous observations, our research group demonstrated that an increased expression of PTX3 was related to diagnosis of prostatic cancer and to an increased risk of prostate cancer development, if assessed in patients undergoing prostate biopsy [36].

Our observations are in line with previous evidences, which indicate that PTX3 could modulate the tumor microenvironment and could be a local or systemic marker of cancer-related inflammation. Increased PTX3 gene expression was reported in ovarian cancer with stromal signature [37], in aggressive breast cancer with distant bone metastases [38–40], in prostate cancer [41], in glioblastoma [42], in anaplastic thyroid carcinoma [43], and in soft tissue liposarcoma [44].

In this context, the close relationship between PTX3 overexpression, tumour microenvironment modulation and complement cascade activation may represent an interesting pathogenic mechanisms, although experimental data on the activation of the complement cascade in renal cancer are limited. PTX3 has been shown to bind C1q as well as MBL inducing the activation of the complement cascade [45].

In our setting, we demonstrated a clear co-localization with C1q, but not with MBL suggesting a local activation of the complement system through the classical pathway. Interestingly, the activation of the complement system in our setting did not lead to the formation of the terminal complement complex with the subsequent lysis of the neoplastic cells. As in most neoplasia, transformed cells may activate several mechanisms to escape complement-dependent lysis. In particular, the production of CD59 or protectin, one of the main inhibitors of C5b-9 assembly ubiquitously expressed at low levels in normal conditions is significantly increased in many tumors [46]. Indeed, we observed a clear overexpression of this protein in the renal cancer tissues. Thus our observation strongly supports the hypothesis that the inhibition of the complement cascade may play a key role in the escape from immunosurveillance of neoplastic cells and might represent a crucial step in the development of clinically evident neoplastic disease. The factors affecting CD59 expression in this setting remain to be clarified, although the inflammatory milieu may represent the answer also for this event, since Bjorge et al. reported that two key pro-inflammatory cytokines, interleukin-1 (IL-1) and tumor necrosis factor alpha, induce the expression of CD59 in human colonic adenocarcinoma cells [47].

More intriguingly, in our study, the increased expression of PTX3 is associated with a partial activation of the classical pathway of the Complement system with an overexpression of C1q and the receptors of C3a and C5a. In renal cancer, as well as in other tumors, the Complement system seems to emerge as a major regulator of cancer immunity. Complement effectors such as C1q, anaphylatoxins C3a and C5a, and their receptors C3aR and C5aR1, have been associated with tolerogenic cell death and inhibition of antitumor T-cell responses through the recruitment and/or activation of immunosuppressive cell subpopulations such as myeloid-derived suppressor cells (MDSCs), regulatory T cells (Tregs), or M2 tumor-associated macrophages (TAMs) [48–50].

Moreover, the receptor of anaphylatoxin C5a (C5aR) has been strongly detected in metastatic renal cell carcinoma and seems to plays a crucial role in cell invasion via the ERK and PI3 kinase pathways [51]. In another tumor, C3aR expression seems to contribute to melanoma carcinogenesis through the inhibition of neutrophils and CD4+T cell response [52]. In recent observations, the anaphylatoxins C3a and C5a seems to significantly contribute to cancer-related inflammation, recruiting myeloid suppressor cells, and promoting IL-1β and IL-17 response in neutrophils, thus enhancing colon carcinogenesis [53–56].

In our setting, both C3a and C5a receptors were dramatically up-regulated in the renal cancer tissue, supporting the hypothesis that the two soluble modulators available *in situ* after the activation of the complement cascade may play a direct or indirect effect on resident cells to sustain carcinogenesis.

The analysis of PTX3 serum levels before nephrectomy revealed that their levels were significantly higher in patients with ccRCC as compared with non-neoplastic patients. Intriguingly, after nephrectomy PTX3 levels significantly lowered, thus strengthening the relationship between intra-tumor PTX3 production and PTX3 serum levels.

When compared to histologic and clinical grading, the basal levels of PTX3 resulted significantly higher in patients with higher Furhman grading (G3-4) and with both lymph nodes positive distant metastases already present at time of diagnosis, thus suggesting a possible role of PTX not only as diagnostic marker but also as disease severity parameter.

Lastly, if related to patient survival, higher PTX3 serum levels at time of nephrectomy were associated with a significantly lower long-term survival, and shorter time to progression as shown by the Kaplan Meyer curves and confirmed by Cox regression analysis. Data from the cancer genome atlas (TCGA) clear cell renal cell carcinoma patient cohort (KIRC), confirmed our findings showing a reduced survival in patients with high expression levels of PTX3 (Supplementary Figure 1).

Our data seems to be in line with previous studies in other clinical settings. Increased circulating levels of PTX3 were observed in myeloproliferative neoplasms [57], lung cancers [58, 59], soft tissue sarcomas [60], gliomas [61], pancreatic and hepatocellular carcinomas [62, 63]. Moreover, high PTX3 levels were associated with advanced clinical stage and poor overall survival of patients with pancreatic carcinoma [61].

Taken together, our data support the potential role of serum PTX3 as a diagnostic and prognostic marker of ccRCC. Moreover, the strong involvement of complement system in the ccRCC microenvironment strongly support the idea that PTX3 up-regulation modulates the effector routes associated with the cancer-immunity cycle, providing the rationale for new therapeutic combinations aimed to enhance the antitumor efficacy of anti-PD-1/PD-L1 checkpoint inhibitors in this neoplasia.

Our study limits are the monocentric retrospective analysis and the rather limited number of cases. However, further prospective multicenter studies are warranted to confirm our observations.

Taken together, our results suggest that expression of PTX3 can modulate the immunoflogosis in the ccRCC microenvironment, by activating the classical pathway of complement system (C1q) and releasing pro-angiogenic factors (C3a, C5a). The up-regulation of CD59 also inhibits the complement-mediated cellular lysis. Moreover, the finding of elevated serum PTX3 levels in the ccRCC patient before nephrectomy suggests its potential role as biomarker of ccRCC diagnosis and prognosis.

## MATERIALS AND METHODS

### Gene set enrichment analysis (GSEA)

Clear cell-RCC transcriptome data derived from exon array analysis of 20 total samples (10 ccRCC tumor sample and their matched non-tumor kidney tissues samples) were used. Exon array data are deposited in GEO at Series accession number GSE47032. GSEA [64] was used to determine which pathways were statistically enriched across the renal cancer dataset. The normalized enrichment score (NES) was used to evaluate the extent and direction of enrichment of each pathway.

### Analysis of biological networks

Pathway analysis was performed using Ingenuity Pathway Analysis (IPA; Qiagen). The data were obtained from the GSE47032 array and the gene IDs and fold-changes were imported into IPA software. Gene symbols were mapped to their corresponding gene object in the Ingenuity Pathways Knowledge Base (IKB). The networks identified are presented in maps showing interactions between genes. Genes are represented as nodes in the networks. The intensity of the node color indicates the degree of up- or downregulation (upregulation in red, downregulation in green). Canonical pathway analysis was used to identify the signaling pathways, which were most significant in the analyzed data set.

### Cell lines

Three different tumor renal cell lines (RCC-SHAW, RCCBA85#21, primary RCC cells) were tested and cultured in a Roswell Park Memorial Institute medium (RPMI) (Sigma Aldrich, Saint Louis, MO USA), supplemented with 10% fetal bovine serum (FBS), 2 mM l-glutamine, and 100 U/ml penicillin–streptomycin (all from Sigma-Aldrich), and incubated for 48 hours at 37 °C, 5% CO_2_, as previously described [65]. As control lines, human HK-2 cells, a proximal tubular epithelial cell line of human origin, were grown in Dulbecco’s modified Eagle’s medium (DMEM)–F12 medium (Sigma-Aldrich) supplemented with 10% fetal bovine serum (FBS), 2 mM l-glutamine, and 100 U/ml penicillin–streptomycin (all from Sigma-Aldrich) at 37 °C in a humidified atmosphere with 5% CO_2_ [64].

### Study population and tissue collection

30 primary renal tumors were collected from patients who underwent nephrectomy for ccRCC at Urology Unit of University Hospital “Ospedali Riuniti” of Foggia. Detailed clinical and pathological characteristics of the patients are summarized in Supplementary Table 1. A control group of 10 subject underwent renal biopsy at Nephrology Unit of University Hospital “Ospedali Riuniti” of Foggia in suspicion of chronic nephropathy, but with a normal renal histology, were also analyzed.

Two pathologists confirmed the presence of ccRCC in the neoplastic tissues and excluded tumor cells in the healthy specimens. Tumor and normal tissues were collected, frozen at −80°C according to a standard procedure and stored.

In addition, serum samples were collected from 168 patients who underwent radical or partial nephrectomy for ccRCC and 40 volunteers with no evidence of malignancy at Urology Unit of University Hospital “Policlinico” of Bari. Serum samples were obtained from each patient at the time of nephrectomy and stored at -30°C. Detailed clinical and pathological characteristics of the patients are summarized in Table 1. All patients were preoperatively staged by thoraco-abdominal Computed Tomography or Magnetic Resonance Imaging. Tumor staging was reassigned according to the seventh edition of the AJCC-UICC TNM classification. The 2016 World Health Organization and Fuhrman classifications were used to attribute histological type and nuclear grade, respectively. Written informed consent to take part was given by all participants. The protocol for the research project has been approved by the local Ethics Committee (Decision n. 152/CE/2014 of September 03, 2014; Ethical Committee at the University Hospital “Ospedali Riuniti” of Foggia).and conforms to the guidelines laid down by the Regional Ethics Committee on human experimentation and to the provisions of the Declaration of Helsinki in 1995.

### PTX-3 serum level assessment

PTX-3 serum levels were tested on serum samples drawn at the time of nephrectomy in the whole study population. Circulating PTX3 was measured was assayed using a commercially available ELISA Kit, according to the manufacturer's instructions (R&D Systems, Minneapolis, MN), as previously described [66, 67].

### Indirect immunofluorescence and confocal laser scanning microscopy

A double-label immunofluorescence was performed to evaluate the expression of PTX-3, C1q, MBL, C3aR, C5R1, C5b-9 and CD59 and their eventual co-localization. To this purpose we employed the following primary antibodies: rat monoclonal IgG2a anti-PTX-3 antibody (clone MNB4, Abcam, Cambridge UK), mouse monoclonal IgG2b anti-C1q (clone JL-1; Abcam); rabbit monoclonal IgG anti-Mannose Binding Lectin (anti-MBL) (clone EPSISR5; Abcam); rabbit polyclonal IgG anti-C3aR (Abcam); mouse monoclonal IgG2a anti-C5R1/CD88 (clone P12/1; Abcam); mouse monoclonal IgG2a anti-C5b-9 (clone aE11; Abcam); rabbit polyclonal IgG anti-CD59 (Sigma-Merck KGaA, Darmstadt, Germany).

Frozen tissue sections were incubated at 4°C over night with a mixture of primary antibodies diluted 1:100 in PBS pH 7.4. The immune complexes were detected using the Alexa-Fluor 488 goat anti-rat and 546 goat anti-mouse IgG and 546 goat anti-rabbit IgG (all from Alexa, Thermo Fisher, Waltham, MA).

After washing in PBS (3x5’) the sections and the negative control were incubated 1h at room temperature with goat anti-rat IgG 488 and goat anti-mouse IgG 546 or goat anti-rabbit IgG 546, as appropriate. All secondary antibodies were used at a dilution of 1:250.

To stain the nuclei, after washing in PBS pH 7.4 (3×5’) samples were incubated with TO-PRO diluted 1:5000 in PBS pH 7,4 (Invitrogen-Molecular Probe, Thermo Fisher, Waltham, MA). The slides were mounted in Gel Mount (Sigma) and sealed.

Specific fluorescence was evaluated by confocal microscopy using the Leica TCS SP5 (Leica, Wetzlar, Germany) equipped with argon-krypton (488 nm), green-neon (543 nm), and helium-neon (633 nm) lasers. Fluorescence quantification was performed as previously described [68, 69].

### Statistical analysis

Statistical analysis was performed as described elsewhere [69, 70]. In detail, statistical calculations were performed with MedCalc 9.2.0.1 (MedCalc software, Mariakerke, Belgium) and PASW 18 software (PASW 18, SPSS, Chicago, Ill, USA). Comparisons of median protein values between different groups were evaluated by Mann–Whitney U test. Receiver Operating Characteristic (ROC) curve analysis was performed to identify the PTX3 cut-off for survival stratification.

In the cancer-specific survival (CSS) analysis, patients who died of RCC unrelated causes or were lost to follow-up were censored. Progression-free survival (PFS) was calculated from the date of surgery to the date of disease recurrence. Estimates of CSS and PFS were calculated according to the Kaplan–Meier method and compared with the log-rank test. Univariate and multivariate analyses were performed using the Cox proportional hazards regression model to identify the most significant variables for predicting CSS and PFS. A backward selection procedure was performed with removal criterion P > 0.10 based on likelihood ratio tests. A P-value of < 0.05 was considered statistically significant.

### Ethics approval

The present study involving human participants was approved by the local ethical committee (Decision n. 152/CE/2014 of September 03, 2014; Ethical Committee at the University Hospital “Ospedali Riuniti” of Foggia). All procedures performed the present study were in accordance with the ethical standards of the Declaration of Helsinki and all the enrolled patients provided an informed consent to participate to the present study.

## Supplementary Material

Supplementary Figure 1

Supplementary Table 1
