# Supplementary material for: PTX3 modulates the immunoflogosis in tumor microenvironment and is a prognostic factor for patients with clear cell renal cell carcinoma
Source: Aging (Albany NY). 2020 Apr 28;12(8):7585–602. doi: 10.18632/aging.103169 (PMC7202504; doi:10.18632/aging.103169)
Supplement: Supplementary Figure 1 [file aging-12-103169-s002..pdf]

SUPPLEMENTARY FIGURE

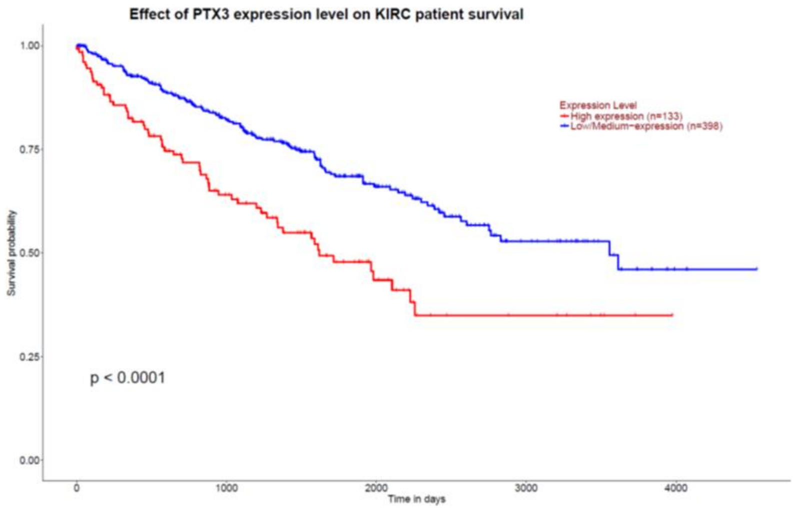

Supplementary Figure 1. Kaplan-Meier survival curves, stratified by the expression level (FPKM) of PTX3 in the tumor tissue at the time of diagnosis. Data from the cancer genome atlas (TCGA) clear cell renal cell carcinoma patient cohort (KIRC).
