# Supplementary material for: PTX3 modulates the immunoflogosis in tumor microenvironment and is a prognostic factor for patients with clear cell renal cell carcinoma
Source: Aging (Albany NY). 2020 Apr 28;12(8):7585–602. doi: 10.18632/aging.103169 (PMC7202504; doi:10.18632/aging.103169)
Supplement: Supplementary Table 1 [file aging-12-103169-s001..pdf]

SUPPLEMENTARY TABLE

Supplementary Table 1. Clinical and pathological characteristics of patients with renal clear cell carcinoma subjected to PTX3 tissue expression analysis.

**Clinical characteristics of RCC patients**		
Patients, n		30
Age, n		59.1±11.1
Female Gender, n (%)		13 (43.3%)
Diabetes Mellitus, n (%)		11 (36.7%)
C reactive protein (CRP), mg/dL		4.5±1.5
CKD-EPI eGFR, ml/min/1.73 m^2		94.5±10.5
**Histologic characterization of of RCC patients**		
Furhman Grading	G 1	5 (16.7%)
	G 2	18 (60.0%)
	G 3	4 (13.3%)
	G 4	3 (10.0%)
TNM/AJCC Staging	pT1a	5 (16.7%)
	pT1b	13 (43.3%)
	pT2a	5 (10.0%)
	pT2b	3 (10.0%)
	pT3a	4 13.3%)
	pT3b-c	0 (0.0%)
	pT4	0 (0.0%)

Abbreviations: CKD-EPI, Chronic Kidney Disease EPIdemiology Collaboration; eGFR, estimated Glomerular Filtration Rate; TNM/AJCC: Tumor size, Lymph Nodes affected, Metastases/American Joint Committee on Cancer.
Values are expressed as mean ± standard deviation, or number of cases and (percentage).
